# Considerations and quality controls when analyzing cell-free tumor DNA

**DOI:** 10.1016/j.bdq.2018.12.003

**Published:** 2019-02-13

**Authors:** Gustav Johansson, Daniel Andersson, Stefan Filges, Junrui Li, Andreas Muth, Tony E. Godfrey, Anders Ståhlberg

**Affiliations:** a Sahlgrenska Cancer Center, Department of Pathology and Genetics, Institute of Biomedicine, Sahlgrenska Academy at University of Gothenburg, Medicinaregatan 1F, 413 90, Gothenburg, Sweden; b Wallenberg Centre for Molecular and Translational Medicine, University of Gothenburg, Gothenburg, Sweden; c Respiratory Inflammation and Autoimmunity, IMED Biotech Unit, AstraZeneca, Gothenburg, Sweden; d Department of Surgery, Institute of Clinical Sciences, Sahlgrenska Academy at the University of Gothenburg, Gothenburg, Sweden; e Department of Surgery, Boston University School of Medicine, 700 Albany Street, Boston, MA, 02118, USA; f Department of Clinical Pathology and Genetics, Sahlgrenska University Hospital, 413 45, Gothenburg, Sweden

**Keywords:** Plasma, Cell-free DNA, Cell-free tumor DNA, DNA barcoding, Liquid biopsy, Mutation detection, Quality controls, Sample preprocessing, SiMSen-Seq

## Abstract

Circulating cell-free tumor DNA (ctDNA) is a promising biomarker in cancer. Ultrasensitive technologies enable detection of low (< 0.1%) mutant allele frequencies, a pre-requisite to fully utilize the potential of ctDNA in cancer diagnostics. In addition, the entire liquid biopsy workflow needs to be carefully optimized to enable reliable ctDNA analysis. Here, we discuss important considerations for ctDNA detection in plasma. We show how each experimental step can easily be evaluated using simple quantitative PCR assays, including detection of cellular DNA contamination and PCR inhibition. Furthermore, ctDNA assay performance is also demonstrated to be affected by both DNA fragmentation and target sequence. Finally, we show that quantitative PCR is useful to estimate the required sequencing depth and to monitor DNA losses throughout the workflow. The use of quality control assays enables the development of robust and standardized workflows that facilitate the implementation of ctDNA analysis into clinical routine.

## 1 Introduction

Mutation analysis of liquid biopsies to identify cancer, monitor treatment and detect relapses is an emerging field of research. The presence of circulating cell-free tumor DNA (ctDNA) has been recognized for decades, but lack of suitable analytical technologies has prevented its way into clinical use [1,2]. The major obstacle to analyze ctDNA is that it usually comprises only a minute fraction of the total circulating cell-free DNA (cfDNA) pool [3]. Consequently, mutant allele frequencies ≤ 0.1% need to be reliably detected. Several methods, such as digital PCR [[4], [5], [6], [7], [8]] and COLD-PCR [9], have the required sensitivity but are restricted to single, or few, pre-defined target mutations. Conventional next-generation sequencing (NGS) allows the analysis of multiple target sequences but fails to identify mutations below 1–3% allele frequency. However, NGS methods that correct sequencing errors using barcoding sufficiently improve sensitivity [[10], [11], [12], [13]].

Cell-free DNA, including ctDNA, is released from cells via apoptosis, necrosis, as well as active secretion, and can be found in various body fluids, including blood plasma, urine, sputum, cerebrospinal fluid, pleural fluid, cyst fluid and saliva [14]. Analysis of ctDNA in liquid biopsies includes several biological and technical challenges besides the ability to detect low ctDNA frequencies. The concentration of cfDNA is overall low, which is problematic as many techniques require higher DNA amounts than can be isolated from a typical plasma sample. Healthy individuals rarely have more than 30 ng cfDNA per ml of plasma, with most having less than 10 ng per ml plasma [15,16]. In late-stage malignancies, higher total cfDNA levels are often observed [17,18]. However, elevated levels can occur for other reasons, such as stroke, myocardial infarction, sepsis, inflammation, trauma [19,20] and physical exercise [21], reducing the usefulness of total cfDNA levels in cancer diagnostics. In clinical applications that involve early detection of tumors, the amount of ctDNA is usually negligible compared to cfDNA levels. An additional complexity of assessing plasma DNA is that the molecules are highly fragmented. The most common fragment size of cfDNA is ∼166 bp, which corresponds to DNA wrapped around a nucleosome plus a ∼20 bp linker bound to histone H1 [[22], [23], [24]]. In ctDNA, this linker fragment appears to be trimmed away, resulting in even shorter fragments [18,25]. Accordingly, applied methods must be able to analyze fragmented DNA to achieve high sensitivity.

**Fig. 1:**
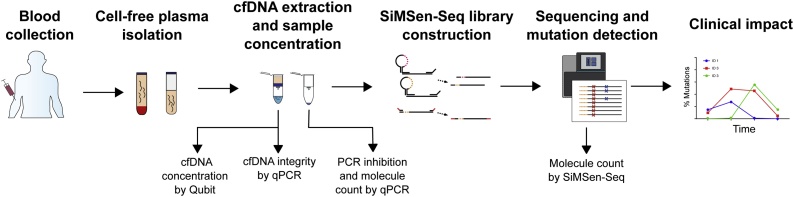
The workflow of liquid biopsy analysis. The quality controls steps applied in this study are indicated by vertical arrows.

In this study, we discuss the liquid biopsy workflow, from sampling to ctDNA analysis using ultrasensitive NGS (Fig. 1), outlining central experimental issues that need to be considered when analyzing ctDNA. We show how simple quality control assays can be applied to evaluate quality and quantity of plasma cfDNA at different experimental steps.

## 2 Results and discussion

### 2.1 Limit of detection and sampling strategies

**Fig. 2:**
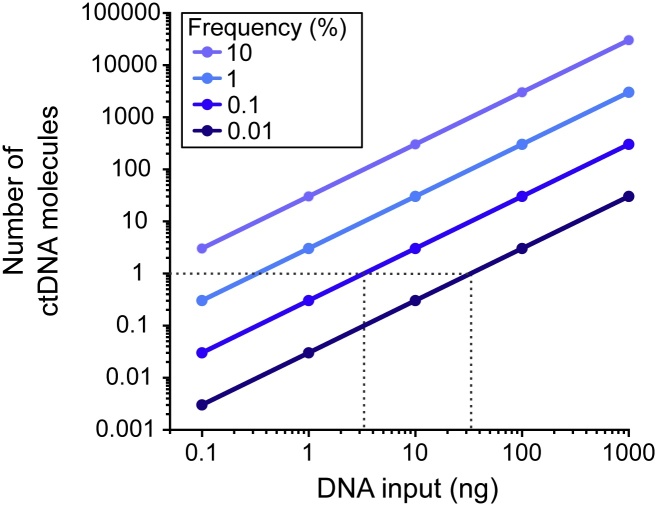
Limit of detection for ctDNA analysis. The ability to detect ctDNA molecules depends on total DNA input. To calculate the relationship between DNA input (ng DNA) and molecule numbers we assumed that the weight of a human haploid genome is $3.3\;\times \;10^{9}bp\;\times \;650Da=\;2.15\;\times \;10^{12}\;Da$, where 650 Da corresponds to the average weight of a base-pair and that one Da equals 1.67 × 10^−24^ g. Consequently, the weight of human genome is $3.59\;\times \;10^{-12}\;g$, i.e., 1 ng human genomic DNA contains about 278 haploid genomes [26,27]. Dashed horizontal line indicate one ctDNA molecule.

Several ultrasensitive methods to analyze ctDNA exist (Table 1). Most NGS based methods use unique molecular identifiers that can be either exogenously attached or inferred from an endogenous sequence context. Other methods reach high sensitivity without the use of unique molecular identifiers and some techniques, such as digital PCR, are not sequencing dependent. Moreover, bioinformatical approaches may also be applied to increase sensitivity. Most ultrasensitive methods report their technical ability to detect a certain mutation frequency. This specification alone is often misleading since it assumes that unlimited amounts of DNA are available. From an experimental point of view, it is more meaningful to determine the limit of detection based on both the applied method and the amount of available DNA. Fig. 2 shows how mutation frequency relates to both total DNA input and number of ctDNA molecules. Several ultrasensitive methods report a sensitivity of ctDNA detection < 0.1%. To utilize the capacity of these approaches ≥ 3.6 ng total cfDNA is needed if we assume that one ctDNA molecule is required for detection, while reaching sensitivity < 0.01% requires 36 ng cfDNA or more. Practically, even more cfDNA is needed since these calculations are based on intact DNA where all molecules can be amplified. Hence, several existing methods will not benefit from further improvements in technology sensitivity when analyzing cfDNA, but rather from other cfDNA workflow improvements. If a plasma sample contains on average one ctDNA molecule, not all samples will be positive even with a method that can detect individual ctDNA molecules. This is due to sampling effects where the probability of finding low number of molecules follows the Poisson distribution.

**Table 1:** Summary of ultrasensitive methods to detect ctDNA.

Method	Detection method	Workflow	Target size	Type of alteration	Sensitivity	Reference
**UMI-based methods, exogenous UMIs**						
**SafeSeqS**	Sequencing	15 cycle barcoding PCR, beads purification, adapter PCR	Targeted	Point mutations, small indels	< 0.1%	[10,28]
**SiMSen-Seq**	Sequencing	2 rounds of PCR followed by beads purification	Targeted	Point mutations, small indels	< 0.1%	[12,13]
**Tam-Seq**	Sequencing	Preamplification, single-plex PCR, barcoding PCR	Targeted	Point mutations, small indels	< 0.1%	[29]
**CypherSeq**	Sequencing	Construction of bacterial vector and estimation of barcode complexity, targeted DNA ligation into vector and PCR amplification	Targeted	Point mutations, small indels	< 0.1%	[30]
**DuplexSeq**	Sequencing	Duplex tag ligation and size selection, adapter PCR	Targeted	Point mutations, small indels	< 0.1%	[11]
**smMIPs**	Sequencing	Probe hybridization, extension and ligation, nuclease treatment and adapter PCR	Targeted	Point mutations, small indels	0.20%	[31]
**UMI-based methods, endogenous UMIs**						
**CircSeq**	Sequencing	DNA shearing, gel extraction, circularize ssDNA, rolling-circle-amplification, purification, adapter ligation, purification, adapter PCR, library purification	Targeted	Point mutations, small indels	< 0.1%	[32]
**Single molecule sequencing**						
**INC-Seq**	Nanopore sequencing	Blunt-end ligation, DNase digestions, rolling circle amplification	Several kilobases	Point mutations, indels, rearrangements	0.1 - 1%	[33]
**CCS SMRTbell**	Sequencing	Template construction by hairpin ligation, ligation control, single-molecule real-time sequencing	Several kilobases	Point mutations, indels, rearrangements	>2.5%	[28,34]
**Non-UMI-based methods**						
**ARMS**	PCR	PCR	SNPs	Known point mutations	< 0.1%	[35]
**BEAMING**	FACS	Emulsion PCR, probe hybridization, magnetic flow detection	SNPs	Known point mutations	< 0.1%	[36]
**CAPP-Seq**	Sequencing	Design selector probes, hybridize probes to sample, enrich for genomic regions covered by selectors	Targeted	Point mutations, indels, rearrangements	< 0.1%	[37]
**Digital PCR**	Digital PCR	Incorporate individual molecules into partitions, amplify material per well/droplet, read fluorescence	SNPs	Known point mutations	single molecule	[4,38]
**Bioinformatical methods**						
**Base-PER**	In silico error correction	Bioinformatics approach where position based error rate is compared to minor allele frequencies of each position	Targeted	Point mutations, small indels	< 0.1%	[39]
**iDES**	In silico error correction	Modeling position-specific errors using a zero-inflated statistical model in a training cohort of control samples to allow error suppression of stereotypical errors in independent samples	Capture	Point mutations, small indels	< 0.1%	[40]
**deepSNV**	In silico error correction	Model nucleotide counts on both strands using hierarchical binomial model and test likelihood ratio for each base	Targeted	Point mutations, small indels	< 0.1%	[41]
**ERASE-Seq**	In silico error correction	Utilize technical replicates in conjunction with background error modeling based on negative binomial distribution	Targeted	Point mutations, small indels	< 0.1%	[42]

UMI, Unique Molecular Identifier; SNP, Single-Nucleotide Polymorphism.

**Fig. 3:**
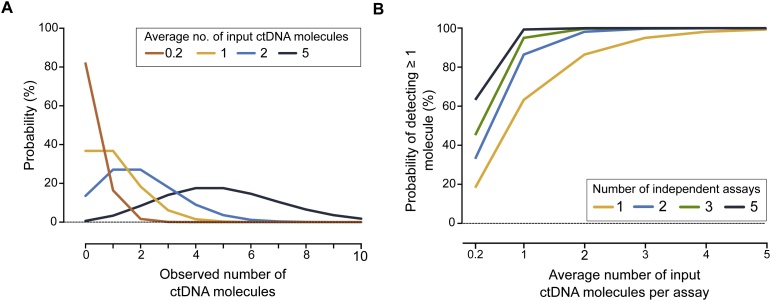
Probability of ctDNA detection due to sampling effects. (A) The probability of detecting a specific number of ctDNA molecules is shown when the average number of input ctDNA molecules is changed, e.g., if analyzed plasma volume is altered. (B) The probability to detect ≥ 1 ctDNA molecule when the average number of ctDNA molecules per sample changes from 0.2 to 5, as well as when the number of assays increases from one to five is shown.

Fig. 3A shows that when on average one ctDNA molecule is added to the analysis there is a 37% risk that no molecule will be present in the analyzed sample. However, even if the average number of molecules is less than one there is still a chance to detect ctDNA. For example, when on average 0.2 ctDNA molecules are sampled there is still an 18% chance to detect it. A universal approach to improve the probability of detecting ctDNA is to increase the volume of plasma analyzed. For instance when a given sample volume contains one ctDNA molecule the probability of not detecting ctDNA can be reduced to 5% if the volume is increased 3-fold.

Another strategy to increase the sensitivity of ctDNA detection is to analyze several mutations simultaneously using multiple assays. Fig. 3B shows the benefit of analyzing multiple and independent mutations when on average 0.2–5 ctDNA molecules are present per assay. The probability of detecting ctDNA increases with the number of assays, where the chance to detect ≥ 1 ctDNA molecule for any assay when on average one ctDNA molecule is present per assay is 95% using three assays and 99.3% applying five assays. The probability of detecting a specific number of ctDNA molecules using multiple assays is shown in Supplementary Figure S1.

### 2.2 Isolation of cfDNA and detection of cellular DNA contamination

Plasma isolation and DNA extraction affect assay sensitivity in terms of both cfDNA yield and quality. To extract cfDNA, plasma should be prepared as fast as possible when using standard EDTA tubes to minimize artifacts. When plasma cannot be prepared within a reasonable time (up to six hours) tubes containing preservatives may be used, such as Cell-Free DNA BCT (Streck) and cf-DNA/cf-RNA preservative tubes (Norgen). These tubes prevent cellular DNA leaking into the plasma for several days. Suboptimal storage condition can however impact the volume of available plasma resulting in an overall lower yield of cfDNA from the samples [43]. Despite potential problems, both EDTA and preservative tubes may provide high-quality cfDNA. To avoid cfDNA degradation, plasma should be stored at −80 °C, but long-term storage (>3 years) may cause reduced cfDNA yield [44].

An optimal cfDNA extraction approach should purify all cfDNA fragments to the same extent, maximize yield and minimize the presence of inhibitors. Today, most methods are based on either magnetic beads or silica-based membranes. Supplementary Table S1 shows an overview of studies where different extraction protocols were tested and compared. Table 2 shows how the cfDNA was assessed after extraction in these studies. It should be noted that various blood collection tubes have different extraction requirements. For example, beads purification with MagMax cell-free DNA isolation kit requires an initial protease treatment using Cell-Free DNA BCT (Streck) plasma, while this pre-treatment should not be performed with plasma isolated from standard EDTA tubes.

**Table 2:** Overview of applied methods to evaluate extracted cfDNA.

**Type of samples**	**Yield**	qPCR	Fluorometer	Digital PCR	Sequencing	Nanodrop	**DNA integrity**	qPCR	Electrophoresis	Digital PCR	**Mutation detection**	qPCR	Digital PCR	ARMS	Sequencing	**Inhibition**	qPCR	**Ref**
Healthy controls	●			●			●			●								[45]
Healthy controls	●	●					●	●										[46]
Healthy and cancer patients	●		●				●		●		●	●						[47]
Cancer patients	●		●				●		●		●		●					[48]
Cancer patients	●		●	●			●	●			●		●					[49]
Cancer patients	●	●									●			●				[50]
Pregnant women	●	●																[51]
Healthy controls	●	●	●		●		●		●		●				●			[52]
Cancer patients	●	●																[53]
Cancer patients	●	●					●	●			●	●	●					[54]
Healthy controls	●	●		●			●	●								●	●	[55]
Pooled sera from patients	●	●	●				●		●									[56]
Healthy and cancer patients	●	●	●				●	●										[57]
Cancer patients	●	●									●	●						[58]
Healthy and cancer patients	●		●	●		●												[59]
Healthy controls	●	●																[60]
Healthy controls	●	●					●	●										[61]
**Number of studies**	17	12	7	4	1	1	11	6	4	1	7	3	3	1	1	1	1	

qPCR, quantiative PCR; ARMS, Amplification-Refractory Mutation System.

**Fig. 4:**
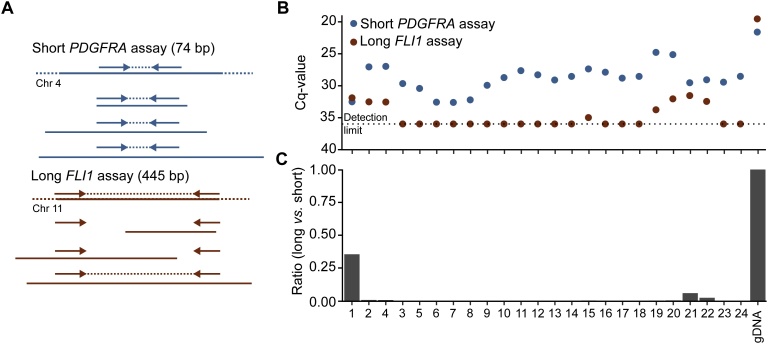
Determination of contaminating cellular DNA using qPCR. (A) Schematic overview of short and long assays used to amplify DNA fragments of different lengths. (B) Cellular DNA contamination assessed by qPCR. Cycle of quantification values (Cq-values) for short *PDGFRA* and long *FLI1* qPCR assays analyzing 24 cfDNA samples and one genomic DNA (gDNA) control. To save clinical material all qPCRs were analyzed using no technical replicates. (C) Degree of cellular DNA contamination. The ratio between long and short qPCR assays was calculated as $2^{\left({Cq}_{short}-{Cq}_{long}-2.1\right)}$ where 2.1 is the ΔCq-value for genomic DNA. Note that the short assay will detect both cfDNA and cellular DNA.

Contamination of ctDNA with cellular DNA will usually not affect general technical performance, rather the opposite since intact DNA is more accessible for amplification. If the amount of ctDNA is reported as the fraction between ctDNA and cfDNA, the ratio will be underestimated due to cellular contamination. If the ctDNA analysis is reported as positive, negative, or as molecules per ml plasma, no such bias is introduced. However, when the amount of contaminating cellular DNA is in the same range or higher than that of cfDNA, assay sensitivity to detect ctDNA may be compromised due to method constraints. Similarly, methods that use a fixed amount of cfDNA as input will also suffer from decreased sensitivity due to cellular DNA contamination. The necessary sequencing depth will increase, and thereby also the sequencing cost. There are different strategies to assess the degree of cellular DNA contamination. DNA capillary electrophoresis can be used which also allows estimation of DNA fragment sizes. Another approach is to quantify short and long DNA fragments using quantitative PCR (qPCR) [62]. The advantage of qPCR is that it generates a quantitative measure of the number of short (cfDNA) and long (cellular DNA) molecules that can be amplified. Fig. 4 shows the degree of contaminating cellular DNA in 24 extracted plasma samples from patients diagnosed with gastrointestinal stromal tumor. Eight samples displayed detectable levels of long (445 bp) DNA fragments using an assays targeting the *FLI1* gene, but only in one sample (Sample 1) was the amount of cellular DNA in the same range as cfDNA.

### 2.3 Assay sensitivity depends on amplicon size and DNA fragmentation

**Fig. 5:**
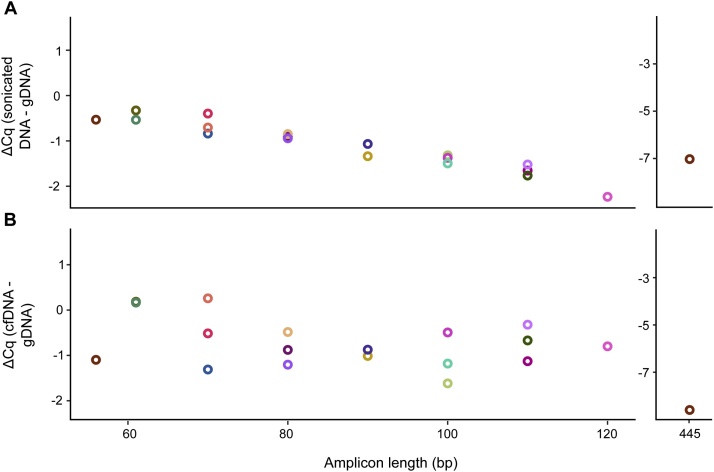
Assay sensitivity depends on DNA fragmentation. The ΔCq-value comparing (A) genomic DNA (gDNA) and sonicated DNA, as well as (B) gDNA and cfDNA. Nineteen qPCR assays with variable amplicon length were used and analyzed as triplicates. The same amount of DNA (1.6 ng) was used in each experiment, where the DNA concentrations had been assessed with a Qubit Fluorometer.

All PCR-based methods, including qPCR, digital PCR, and NGS, are sensitive to DNA fragmentation. To test the effect of assay length we used 19 qPCR assays ranging in amplicon size from 56 bp to 120 bp and one 445 bp long assay (Supplementary Table S2). We analyzed both intact and sonicated genomic DNA from a cell line as well as pooled cfDNA from patients diagnosed with gastrointestinal stromal tumor. The fragment sizes of cfDNA and sonicated DNA are shown in Supplementary Figure S2. Fig. 5A shows that assay sensitivity decreased with increased assay length when DNA had been sonicated. The assay readout of each assay is comparable since the PCR efficiencies of all assays were close to 100% (Supplementary Table S3). Assay sensitivity decreased 31%, 79% and 99% for the 56 bp, 120 bp and 445 bp long assay, respectively. Assay variability is shown in Supplementary Figure S3. Fig. 5B and Supplementary Figure S3B show that assay sensitivity follows the same trend when analyzing cfDNA. However, the trend for the short assays is less clear. For example, two of the 110 bp long assays showed improved performance over the 56 bp long assay. These results can be explained by the fact that cfDNA fragmentation is regulated by nucleosome positioning that depends on the cell type that the cfDNA originates from [63], while sonicated DNA is fragmented more randomly. Altogether, our data show that assay sensitivity is improved by the use of short amplicon lengths. One drawback of using short assays is that the number of assays will be high if large target sequence regions need to be covered.

### 2.4 Detection of sample PCR inhibition

Liquid biopsies contain a plethora of potential PCR inhibitors, such as heparin, hormones, immunoglobulin G and lactoferrin [64,65]. If the blood sample is disturbed before plasma preparation, hemolysis may cause additional release of inhibitors [66]. However, PCR inhibition does not seem to increase with prolonged storage or with the use of preservatives [67]. Sample type and extraction strategy will determine the purity of isolated cfDNA, and there is a trade-off between obtaining high yield and minimizing the presence of inhibitors. Furthermore, to maximize the chance to detect ctDNA, the isolated DNA sample is often concentrated to allow analysis of all available molecules in one single reaction tube. Methods to concentrate cfDNA are based on silica membranes, size filtering membranes, chemical precipitation, or vacuum centrifugation. Potential drawbacks are total yield losses and that PCR inhibitors may become concentrated.

**Fig. 6:**
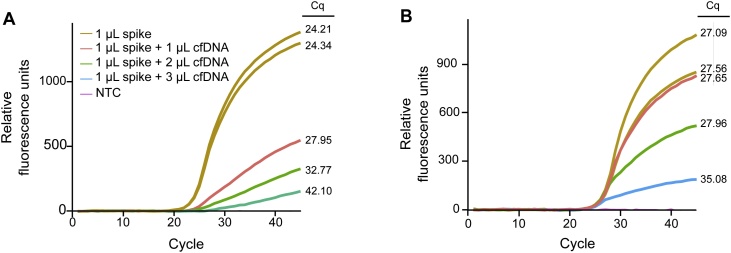
Detection of sample inhibition using qPCR. Three different concentrations of extracted pooled normal human plasma were analyzed. To each sample the same amount of an artificial DNA molecule was added (1 μL spike), including two samples without any cfDNA. The” Short 74 bp *PDGFRA* assay” was used. (A) Cell-free DNA extracted using a silica-membrane method (QIAamp Circulating Nucleic Acid Kit) and concentrated with size limiting membrane (Vivacon 500 MWCO 30,000 Daltons). (B) Cell-free DNA extracted using a magnetic beads method (MagMAX Cell-Free DNA Isolation Kit) with size filtering membrane (Vivacon 500 MWCO 30,000 Daltons). NTC, Negative Template Control.

PCR inhibition may affect NGS performance [68,69]. Several concepts to detect PCR inhibition are developed within the field of qPCR that also are potentially useful in NGS. One such strategy is to analyze cfDNA with a control qPCR assay and evaluate the amplification curve [70,71]. Here, we compared two extraction methods using the same pooled normal human plasma. In Fig. 6A we used a silica membrane-based extraction method, while a magnetic beads-based approach was applied in Fig. 6B. The amplification curves level off more quickly when the amount of cfDNA is increased for both methods, indicating PCR inhibition. We also added an artificial molecule, in excess, that is amplified by the same primer pair as the target amplicon. Hence, it was possible to measure inhibition by comparing Cq-values between samples. In our test, the magnetic beads-based approach was superior to the silica membrane-based method. When we generated sequencing libraries from clinical samples extracted with the method causing PCR inhibition only 26% (n = 15) were successful compared to 97% (n = 93) when samples were extracted with the method without detectable inhibition (data not shown). Sometimes it is possible to rescue a PCR inhibited sample by either re-extraction or dilution. The consequences of inhibition depend on the application. NGS fails if the inhibition is severe, and if data are still generated, they may suffer from different biases including erroneous ctDNA quantification.

**Fig. 7:**
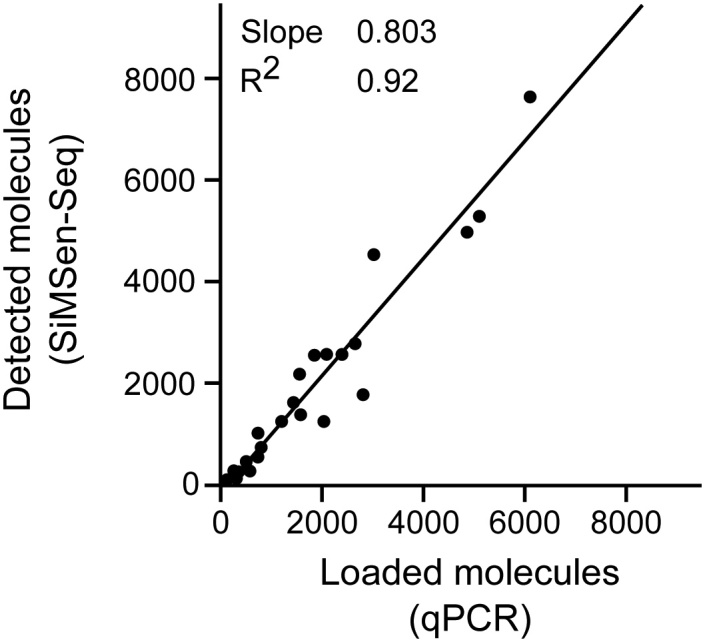
Relationship between SiMSen-Seq and qPCR data. The linear regression between the two methods is shown. The primers of the “Short 74 bp *PDGFRA* assay” were used both for qPCR and SiMSen-Seq. The analyzed samples are the same as analyzed in Fig. 4 (n = 23).

Most ultrasensitive methods, including barcoded NGS and digital PCR, require that the amount of cfDNA used in the reaction is known to achieve optimal assay performance. If cfDNA is sequenced with too low coverage, error-correction is not possible, while the cost increases if the sample is over-sequenced. Here, the inhibition test is also useful since it will quantify the number of amplifiable molecules present in the sample. Fig. 7 shows a linear correlation between the molecule numbers quantified by qPCR and barcoded NGS, i.e., SimSen-Seq. Primers with identical target specific sequence were used in both qPCR and SiMSen-Seq. None of these samples showed any sign of inhibition (Supplementary Figure S4). Consequently, qPCR is conveniently and easily used to estimate the required sequencing depth per sample.

### 2.5 Analysis of cfDNA losses throughout the liquid biopsy workflow

**Fig. 8:**
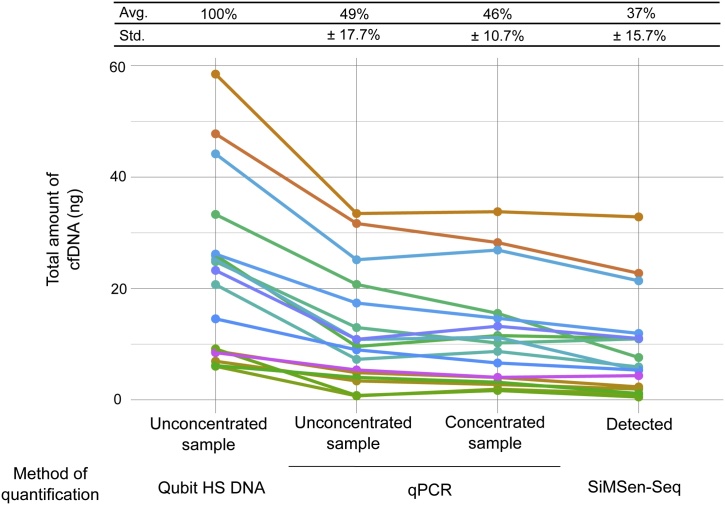
The concentration of cfDNA throughout the experimental workflow. Plasma samples (cf-DNA/cf-RNA tubes, Norgen) were extracted (MagMAX Cell-Free DNA Isolation Kit), concentrated (Vivacon 500 MWCO 30,000 Daltons) and analyzed by SiMSen-Seq. The amount of cfDNA was assessed at different steps using multiple methods. Quantitative PCR data were converted to cfDNA amounts using standard curves of non-fragmented DNA. The “Short 74 bp *PDGFRA* assay” was used for quantification of both unconcentrated and concentrated samples. SiMSen-Seq and qPCR analyses were performed with the same target primers. Molecule numbers generated by SimSen-Seq were converted to cfDNA amounts using the formula in Fig. 2 legend. Total amount of cfDNA was adjusted to compensate for volume loss due to each quality control assay. The analyzed samples are a subset of the samples analyzed in Fig. 4 (n = 17).

All pre-analytical steps (Fig. 1) including blood sampling, extraction, sample concentration, and final ctDNA analysis may influence the overall assay sensitivity. To monitor where losses occur we quantified cfDNA throughout the pre-analytical workflow in 17 of our plasma samples from patients diagnosed with gastrointestinal stromal tumor. Fig. 8 shows that cfDNA yield varies between patient samples and decreases throughout the experimental workflow. The amount of extracted cfDNA was analyzed with Qubit Fluorometer, then qPCR was performed to estimate losses due to fragmentation and sample concentration. Finally, cfDNA was sequenced with SiMSen-Seq. Forty-nine percent of the extracted cfDNA was amplifiable by qPCR. The sample concentration step showed a minor 3% loss of molecules and an additional 9% loss was observed in the SiMSen-Seq step. Thirty-seven percent of the initial molecules were quantified by SiMSen-Seq. The molecule loss due to fragmentation agrees with the data in Fig. 5. However, our analyses do not assess the loss in the extraction step. Numerous studies have compared the cfDNA yield between extraction methods (Supplementary Table S1), but the absolute loss is difficult to estimate. Synthetic molecules with known concentrations may be spiked into the plasma to monitor the process [55], but it is difficult to completely mimic true cfDNA and its sample matrix [72].

Quality control assays are useful tools to evaluate, compare and optimize liquid biopsy workflows. These tests are also useful for standardized workflows since they will reveal information about PCR inhibition, genomic DNA contamination and the amount of cfDNA that can be amplified in individual samples. The main disadvantages with quality control assays are that they consume time, resources, including some of the limited cfDNA. In our analyses (Fig. 8) we used ∼6% of the unconcentrated cfDNA and ∼16% of the concentrated cfDNA. Practically, when workflows are being developed and optimized, it is beneficial to use multiple quality controls, while fewer can be applied when analyzing samples with standardized protocols. In our experience, the most important quality control is to quantify the cfDNA that is loaded into the barcoded NGS, since it will impact the required sequencing depth and indicate the presence of sample inhibitors.

## 3 Materials and methods

### 3.1 Blood sampling and cfDNA extraction

Peripheral blood was collected in cf-DNA/cf-RNA Preservative Tubes (Norgen) from patients diagnosed with gastrointestinal stromal tumors treated at the Department of Surgery, Sahlgrenska University Hospital, Gothenburg, Sweden. The study was approved by the Regional Ethics Authority in Gothenburg (# 485–16 and T795–16). Plasma was isolated according to manufacturer’s recommendations using a single centrifugation (430 x g) at room temperature for 20 min and stored at -80 °C before extraction. For the PCR inhibition test Pooled Normal Human Plasma (Innovative Research) was used. Before cfDNA extraction, samples were thawed and centrifuged (16,000 x g) at 4 °C for 10 min. Five milliliter plasma was extracted using MagMax cfDNA isolation kit (Applied Biosystems) according to the manufacturer’s instructions, and cfDNA was eluted in 75 μL elution buffer. For the PCR inhibition test and the comparison between inhibited and non-inhibited clinical samples, the QIAamp Circulating Nucleic Acid Kit (Qiagen) with 150 μL elution volume was used. Cell-free DNA concentration was quantified using a Qubit Fluorometer version 3 with Qubit dsDNA HS Assay Kits (both Invitrogen). DNA samples analyzed with Fragment Analyzer used the High Sensitivity NGS analysis kit (both Advanced Analytic Technology) according to manufacturer’s instructions.

Extracted cfDNA was concentrated using Vivacon 500 spin columns with 30,000 MWCO Hydrosart membrane (VivaProducts). Here, samples were centrifuged at 5000 x g in room temperature for 15 min and eluted by reversing the column and spinning 2 min at 2500 x g. If the eluted volume was less than 6 μL, nuclease-free water (Invitrogen) was added to the sample to compensate.

### 3.2 Cellular DNA

Control DNA from MDA-MB-231 and T47D breast cancer cell lines (ATCC) were extracted using QIAamp DNA Blood Mini Kit (Qiagen) according to the manufacturer’s recommendation. DNA concentration was quantified with Qubit dsDNA HS Assay Kit. DNA sonication was performed with a Bioruptor Pico in a 0.65 mL tube (both Diagenode) containing 1 μg DNA in 100 μL nuclease-free water. Thirty cycles of sonication, on for 30 s followed by off for 30 s, at 4 °C was applied.

### 3.3 Quantitative PCR

Quantitative PCR was performed in a CFX384 Touch Real-Time PCR Detection System (Bio-Rad) in 10 μL reactions containing 1 × TATAA SYBR GrandMaster Mix (TATAA Biocenter), 400 nM of each primer (desalted, Sigma-Aldrich, see Supplementary Table S2) and 1–2 μL of cfDNA. The following temperature profile was used: 3 min at 98 °C, followed by 50 cycles of amplification (98 °C for 10 s, 60 °C for 30 s, 72 °C for 20 s). A melting curve was included ranging from 60 °C to 95 °C, 1 s per 0.5 °C increment. All primer sequences were checked with Primer-BLAST [73], and all assays were confirmed to be specific using a Fragment Analyzer. All samples were tested using melting curve analysis and all experiment contained no-template control without any amplification of specific PCR products. Quantitative PCR performance was determined with standard curve analysis using a 5-fold dilution of T47D cell line DNA ranging from 60 ng to 0.096 ng (Supplementary Table S3). Cycles of quantification (Cq) values were determined with regression using Bio-Rad CFX Maestro 1.1 (Bio-Rad) software. A Cq-value of 35 was used as the cut-off. Missing data were replaced with a Cq-value of 36. The ratio was set to zero in Fig. 4C, if no specific PCR product was detected for the *FLI1* assay.

In the inhibition test (data shown in Fig. 6) equal amount of a synthetic DNA standard, amplified by the same set of primer that target the cfDNA sequence, was added to all samples to serve as amplification control and Cq-values were determined using a threshold. To quantify the number of amplifiable fragments (data shown in Fig. 7, Fig. 8) a standard curve with 3-fold dilutions ranging from 20 ng to 0.25 ng MDA-MB-231 cell line DNA was analyzed in triplicate.

### 3.4 SiMSen-Seq

Sequencing libraries were constructed using SiMSen-Seq as described [12]. Briefly, 5 μL of cfDNA was barcoded in a 10 μL reaction, containing 1× Phusion High-Fidelity Buffer, 0.2 U Phusion HF polymerase (both Thermo Fisher Scientific), 0.2 mM dNTP (Sigma-Aldrich), 40 nM of each primer (PAGE-purified, IDT) (Supplementary Table S4), 0.5 M l-carnitine inner salt (Sigma-Aldrich). A T100 Thermal cycler (Bio-Rad) was used with the following thermal program, 30 s at 98 °C, followed by 3 cycles of amplification (98 °C for 10 s, 62 °C for 6 min, 72 °C for 30 s), and finished with 15 min at 65 °C and 15 min at 95 °C. Twenty microliters of 45 ng/μL *Streptomyces griseus* protease (Sigma-Aldrich) solution dissolved in TE-buffer solution, pH 8.0 (Thermo Fisher Scientific) was added to each well at the start of the 15 min incubation step at 65 °C to attenuate and degrade the polymerase, reducing the formation of primer dimers. Ten microliter of the barcoded product was then amplified using indexed Illumina adaptor primers (desalted, Sigma-Aldrich) in a 40 μL reaction containing, 1 × Q5 Hot Start High-Fidelity Master Mix (New England BioLabs) and 400 nM of each primer (Supplementary Table S5) using the following thermal program on a T100 Thermal cycler; 98 °C for 3 min, 30–40 cycles of amplification (98 °C for 10 s, 80 °C for 1 s, 72 °C for 30 s, 76 °C for 30 s, all with ramping at 0.2 °C/s. Libraries were purified using the Agencourt AMPure XP system (Beckman Coulter) according to the manufacturer’s instructions at a beads-to-sample ratio of 1. Library quality and quantification were assessed on a Fragment Analyzer using the HS NGS analysis (DNF-474, Advanced Analytical).

Sequencing was performed on a MiniSeq using a High Output Reagent Kit (150-cycles) containing a 20% PhiX Control v3 (all Illumina) with clustering at 0.8 pM. Data were analyzed using Debarcer as described [12,13]. At least three reads with the same barcode (consensus 3) were required to form a valid barcode family.

### 3.5 Statistics

Poisson probabilities were calculated using the probability mass function of the Poisson distribution *P(k)* = *exp*^−λ^ × *λ^k^* / *k!*, where *P(k)* is the probability of observing *k* molecules given an average concentration of *λ* molecules per reaction. All calculations were implemented in R (Version 3.5.1) using the function dpois.

## Conflict of interest statements

GJ is employed by AstraZeneca. AS declares stock ownership in TATAA Biocenter. TEG and AS are co-inventors of SiMSen-Seq that is patent pending.
