# Supplementary material for: Considerations and quality controls when analyzing cell-free tumor DNA
Source: Biomol Detect Quantif. 2019 Feb 13;17:100078. doi: 10.1016/j.bdq.2018.12.003 (PMC6416156; doi:10.1016/j.bdq.2018.12.003)
Supplement: Supplementary file 1 [file mmc1.pdf]

Appendix A - Supplementary Figures and Tables

Considerations and quality controls when analyzing cell-free tumor DNA

Gustav Johansson[1,2,3], Daniel Andersson[1], Stefan Filges[1], Junrui Li[1], Andreas Muth[4], Tony E. Godfrey[5], Anders Ståhlberg[1,2,6*]

[1] Sahlgrenska Cancer Center, Department of Pathology and Genetics, Institute of Biomedicine, Sahlgrenska Academy at University of Gothenburg, Medicinaregatan 1F, 413 90 Gothenburg, Sweden.

[2] Wallenberg Centre for Molecular and Translational Medicine, University of Gothenburg, Gothenburg, Sweden.

[3] Respiratory Inflammation and Autoimmunity, IMED Biotech Unit, AstraZeneca, Gothenburg, Sweden.

[4] Department of Surgery, Institute of Clinical Sciences, Sahlgrenska Academy at the University of Gothenburg, Gothenburg, Sweden

[5] Department of Surgery, Boston University School of Medicine, 700 Albany Street, Boston, MA 02118, USA

[6] Department of Clinical Pathology and Genetics, Sahlgrenska University Hospital, 413 45 Gothenburg, Sweden.

* corresponding author: Anders Ståhlberg, Sahlgrenska Cancer Center, University of Gothenburg, Box 425, 405 30 Gothenburg, Sweden; Tel: +46 31 7866735; anders.stahlberg@gu.se

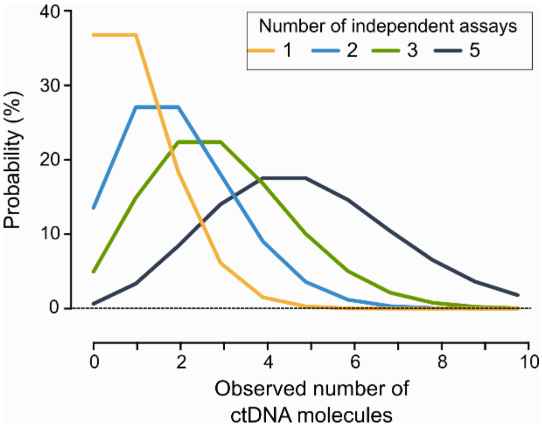

Figure S1. Probability of detection when using multiple assays. *The probability to detect a specific number of ctDNA molecules is shown when the number of mutations analyzed is increased from one to five. The average number of input ctDNA molecules per assay is one.*

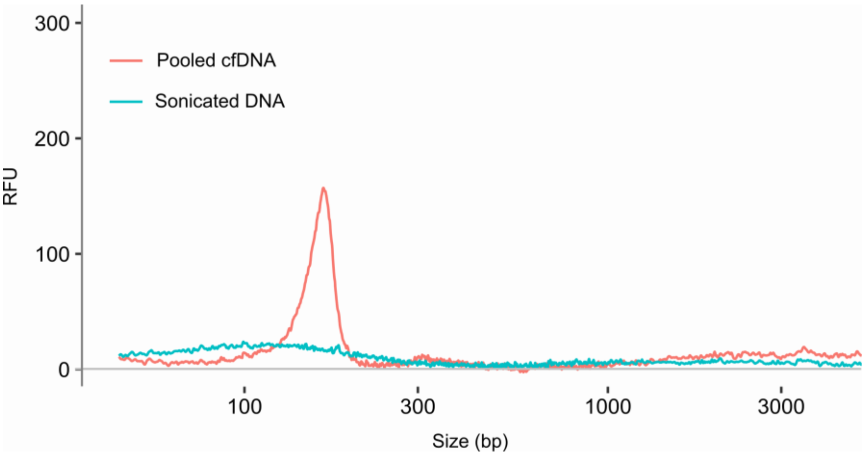

Figure S2. Determination of DNA fragmentation. Fragmentation analysis using Fragment Analyzer with the High Sensitivity NGS Fragment Analysis Kit. Pooled cfDNA from >20 patients diagnosed with gastrointestinal stromal tumors extracted using a magnetic beads method (MagMAX Cell-Free DNA Isolation Kit) and concentrated with size limiting membrane (Vivacon 500 MWCO 30,000 Daltons). Sonicated DNA is generated from MDA-MB-231 breast cancer cell line.

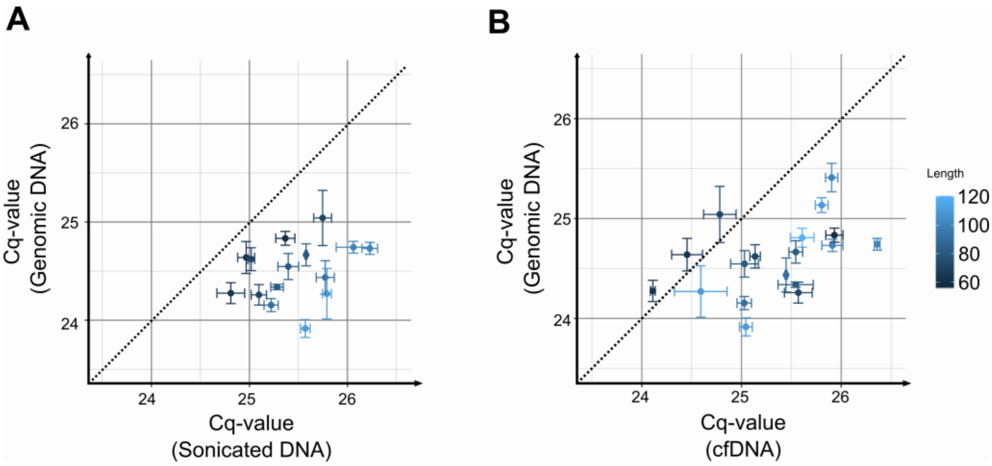

Figure S3. Assay performance depends on DNA fragmentation and amplicon length. Cycle of quantification (Cq)-value comparing **(A)** genomic DNA and sonicated DNA, as well as **(B)** genomic DNA and cfDNA. Nineteen qPCR assays with variable amplicon length were used and analyzed as triplicates. The same amount of DNA (1.6 ng) was used in each reaction, where the DNA concentrations were assessed with a Qubit Fluorometer. Compared to Figure 5 these subplots show standard deviation of assay performance.

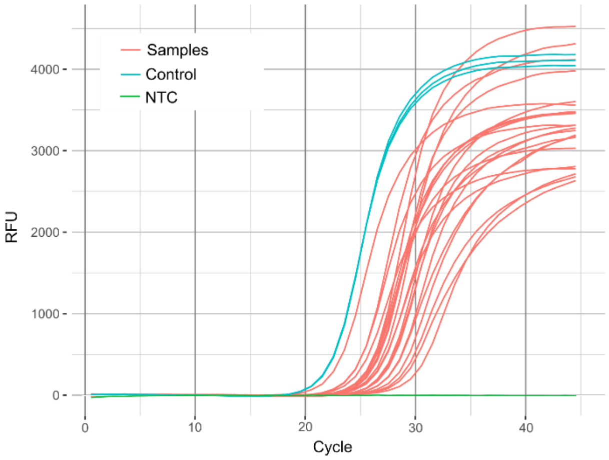

Figure S4. PCR inhibition test. Quantitative PCR evaluation of 24 patient samples diagnosed with gastrointestinal stromal tumors extracted using a magnetic beads method (MagMAX Cell-Free DNA Isolation Kit) and concentrated with size limiting membrane (Vivacon 500 MWCO 30,000 Daltons) Daltons). Control refers to genomic cell lines DNA.

Table S1. Summary of studies comparing different cfDNA extraction protocols.

	References															
	[45]	[47]	[48]	[49]	[50]	[51]	[52]	[53]	[54]	[55]	[56]	[57]	[58]	[59]	[74]	[61]
**Silica or column-based extractions**																
QIAamp circulating Nucleic Acids Kit (Qiagen)	●		●	●	●		●		●	●		●	●	●		●
QIAamp DNA Blood Mini Kit (Qiagen)							●	●		●	●		●		●	●
NucleoSpin Plasma XS Kit (Macherey-Nagel)							●			●		●				
FitAmp Plasma/Serum DNA Isolation Kit (EpiGentek)							●			●						
Plasma/Serum Cell-Free Circulating DNA Purification Midi Kit (Norgen Biotek Corp)	●											●				
Genomic Mini AX Body Fluids (AA Biotechnology)													●			
NucleoSpin PlasmaF Kit (Macherey-Nagel)								●								
QIAamp DNA Mini Kit (Qiagen)		●														
QIAamp DSP virus Kit (Qiagen)						●										
QIAamp UltraSens Virus Kit (Qiagen)																●
QIAamp Virus Spin Kit (Qiagen)															●	
Quick-cfDNA Serum & Plasma Kit (Zymo Research)														●		
Plasma/Serum Cell-Free Circulating DNA Purification Mini Kit (Norgen)												●				
Sherlock AX (AA Biotechnology)													●			
ZR serum DNA Kit (Zymo Research)											●					
**Magnetic beads-based extractions**																
Maxwell RSC ccfDNA Plasma Kit (Promega)	●	●	●	●					●							
ChargeSwitch gDNA Serum Kit, 0.2-1 mL (Invitrogen)											●				●	
Agencourt Genfind Blood and Serum Genomic DNA Isolation Kit (Agencourt Bioscience Corporation)															●	
Chemagic NA Extraction Kit (Perkin-Elmer)												●				
COBAS AmpliPrep Total Nucleic Acid Isolation Kit (Roche)						●										
QIAsymphony Circulating NA Kit (Qiagen)														●		
QIAsymphony DSP Virus/ Pathogen Midi Kit (Qiagen)					●											●
EpiQuick Circulating Cell- Free DNA Isolation Kit (EpiGentek)				●												
Mag-Bind Circulating DNA (Omega Bio-Tek)	●															
MagMAX Cell-free DNA Isolation Kit (Life Technologies)	●															
MagNA Pure LC DNA Isolation Kit - Large Volume (Roche)								●								
MagNA Pure Compact Nucleic Acid Isolation Kit I (Roche)			●											●		
NEXTprep-Mag cfDNA Isolation Kit (Bioo Scientific)				●												
Circulating Cell Free DNA Kit (NeoGeneStar)	●															
**Other extractions**																
Phenol-chloroform											●	●				
Sodium iodide method											●					
Guanidine-resin method											●					
Puregene DNA purification System Cell and Tissue Kit (Gentra)											●					
PME free-circulating DNA Extraction Kit (Analytik Jena)	●			●	●									●		

Table S1. Quantitative PCR primers.

Gene	Ensemble ID	Forward primer [5´ - 3´]	Reverse primer [5´ - 3´]	Amplicon length (bp)	Chr	Forward start	Forward stop	Reverse start	Reverse stop
BAP1	ENSG00000163930	GTCTTGGTCTCCACTAGGGC	GGGCCTTGTCTGTCCACTC	56	3	52406924	52406905	52406869	52406887
DDR2	ENSG00000162733	ACTTAAATAGGGCAAGTTCACTACA	CCACAAAGTAACCCCAAAGGC	61	1	162778570	162778594	162778630	162778610
ERBB2	ENSG00000141736	TACCTTTCTACGGACGTGGG	TCACCTCTTGGTTGTGCAGG	61	17	39711933	39711952	39711993	39711974
AKT	ENSG00000142208	TTCTTGAGGAGGAAGTAGCGT	CTCTCACCACCCGCACG	70	14	104780171	104780191	104780240	104780224
BAP1	ENSG00000163930	CCTTAGTGCAGTGCGGACC	AAAGAGCCGGCCTGTGATA	70	3	52407280	52407262	52407211	52407229
ROS1	ENSG00000047936	CCCCAATACATTATCCTGGAACT	CCATCCGGGCTTTACGCA	70	6	117317217	117317195	117317148	117317165
PDGFRA[1]	ENSG00000134853	GAAGATCTGTGACTTTGGCCTG	GACGTACACTGCCTTTCGAC	74	4	54285897	54285918	54285970	54285951
AKT1	ENSG00000142208	GGTGCCATCATTCTTGAGGAGG	CTCTCACCACCCGCACGTC	80	14	104780161	104780182	104780240	104780222
BAP1	ENSG00000163930	CTTCTTCTCTCCTACAGATTGATGA	CAGCATGGAGATAAAGGTGCAG	80	3	52402691	52402667	52402612	52402633
ERBB2	ENSG00000141736	AATTCCAGTGGCCATCAAAGTG	TGGAGGGGCTTACGTCTAAG	80	17	39723944	39723965	39724023	39724004
BAP1	ENSG00000163930	CTGGCCCGCCCCGAT	ATAGTTTGCAATCTCAGCCTCCAC	90	3	52402913	52402899	52402824	52402847
FCGR3A	ENSG00000203747	CTCGAGCACCCTGTACCATT	CCTGTCCTCACCCCACATTA	90	1	161548623	161548642	161548712	161548693
BAP1	ENSG00000163930	CCCGACCGCAGGATCAAGTAT	AGGAAGAAAGGGCACCTACCTG	100	3	52406333	52406313	52406234	52406255
ESR1	ENSG00000091831	ACAAAGGCATGGAGCATCTG	GGCGCATGTAGGCGGT	100	6	152098734	152098753	152098833	152098818
TP53	ENSG00000141510	GTGGTGAGGCTCCCCTTT	ACTGGGACGGAACAGCTTTG	100	17	7673729	7673746	7673828	7673809
CSMD3	ENSG00000164796	TTCGATGGAGGCAGGACCTA	TGGAACATGGAAGATGGCGA	110	8	112304773	112304792	112304882	112304863
RYR2	ENSG00000198626	ATGGATCTGCAGAAGGATATGGTGG	GCCCATGCTGATCCTTTAAATAAGATAATA	110	1	237781598	237781622	237781707	237781678
SLC26A3	ENSG00000091138	TTGCGATGCCGAAGCAATC	TGGTTGGTGACATTCAGACTT	110	7	107783282	107783300	107783391	107783371
MEK1	ENSG00000169032	TGAGCAGCAGCGAAAGCG	AACACCACACCGCCATTGC	120	15	66435075	66435092	66435194	66435176
FLI[2]	ENSG00000151702	TGAGGCTGAATTATCCACAATGGCTGG	GGGTGTGCCTGCTATGAGAA	445	11	128800759	128800785	128801203	128801184

Primer positions in human reference genome GRCh38/hg38

[1] Used as ”Short assay”.

[2] Used as ”Long assay”.

Table S3 Performance of qPCR primers using standard curves

Assay	PCR Efficiency (%)	Slope	Y-interception	R^2	Std low limit (Cq)
BAP1 (56bp)	100.9%	-3.31 ± 0.04	35.6 ± 0.12	0.998	0.25
DDR2 (61bp)	108.9%	-3.12 ± 0.04	34.0 ± 0.11	0.998	0.04
ERBB2 (61bp)	101.8%	-3.28 ± 0.07	33.9 ± 0.19	0.995	0.53
AKT (70bp)	103.2%	-3.25 ± 0.08	34.2 ± 0.21	0.993	0.13
BAP1 (70bp)	101.4%	-3.29 ± 0.09	35.0 ± 0.26	0.991	0.50
ROS1 (70bp)	105.6%	-3.20 ± 0.04	34.8 ± 0.12	0.998	0.19
PDGFRA (74bp)	107.0%	-3.17 ± 0.03	34.9 ± 0.09	0.999	0.11
AKT1 (80bp)	100.9%	-3.30 ± 0.03	34.7 ± 0.10	0.999	0.11
BAP1 (80bp)	98.2%	-3.37 ± 0.11	35.2 ± 0.33	0.985	1.06
ERBB2 (80bp)	105.6%	-3.19 ± 0.05	33.5 ± 0.14	0.997	0.34
BAP1 (90bp)	111.8%	-3.07 ± 0.06	33.7 ± 0.18	0.995	0.50
FCGR3A (90bp)	103.2%	-3.25 ± 0.03	33.9 ± 0.10	0.999	0.03
BAP1 (100bp)	100.9%	-3.30 ± 0.09	35.0 ± 0.25	0.992	0.70
ESR1 (100bp)	96.8%	-3.40 ± 0.04	35.4 ± 0.11	0.999	0.14
TP53 (100bp)	99.2%	-3.34 ± 0.07	35.2 ± 0.19	0.995	0.41
CSMD3 (110bp)	99.5%	-3.33 ± 0.06	33.5 ± 0.17	0.996	0.52
RYR2 (110bp)	100.4%	-3.32 ± 0.05	34.0 ± 0.15	0.997	0.43
SLC26A3 (110bp)	104.6%	-3.21 ± 0.03	33.8 ± 0.10	0.999	0.06
MEK1 (120bp)	105.6%	-3.19 ± 0.05	33.6 ± 0.15	0.997	0.39
FLI (445bp)	95.0%	-3.44 ± 0.03	32.6 ± 0.10	0.999	0.21

All assays showed a linear dynamic range from 40 ng down to 0.06 ng genomic cell line DNA. For a subset of the assays one replicate in the mid range concentration (1,6 ng) was removed as an outlier. Std low limit = standard deviation of Cq values at the lowest concentration (0.06 ng).

Table S4 SiMSen-Seq primers

Name	Ensable ID	Forward primer [5´ - 3´]	Reverse primer [5´ - 3´]
PDGFRA	ENSG00000134853	GGACACTCTTTCCCTACACGACGCTCTTCCGATCTNNNNNNNNNNNNATGGGAAAG AGTGTCCGAAGATCTGTGACTTTGGCCTG	GTGACTGGAGTTCAGACGTGTGCTCTTCCGATCTGACGTACA CTGCCTTTCGAC

Table S5 Illumina indexing primers

Name	Primer sequence [5' – 3']
Universal forward adapter primer	AATGATACGGCGACCACCGAGATCTACACTCTTTCCCTACACGACGCTCTTCCGATCT
Reverse index 1 adapter primer	CAAGCAGAAGACGGCATACGAGATCGTGATGTGACTGGAGTTCAGACGTGTGCTCTTCCGATCT
Reverse index 2 adapter primer	CAAGCAGAAGACGGCATACGAGATACATCGGTGACTGGAGTTCAGACGTGTGCTCTTCCGATCT
Reverse index 3 adapter primer	CAAGCAGAAGACGGCATACGAGATGCCTAAGTGACTGGAGTTCAGACGTGTGCTCTTCCGATCT
Reverse index 4 adapter primer	CAAGCAGAAGACGGCATACGAGATTGGTCAGTGACTGGAGTTCAGACGTGTGCTCTTCCGATCT
Reverse index 5 adapter primer	CAAGCAGAAGACGGCATACGAGATCACTGTGTGACTGGAGTTCAGACGTGTGCTCTTCCGATCT
Reverse index 6 adapter primer	CAAGCAGAAGACGGCATACGAGATATTGGCGTGACTGGAGTTCAGACGTGTGCTCTTCCGATCT
Reverse index 7 adapter primer	CAAGCAGAAGACGGCATACGAGATGATCTGGTGACTGGAGTTCAGACGTGTGCTCTTCCGATCT
Reverse index 8 adapter primer	CAAGCAGAAGACGGCATACGAGATTCAAGTGTGACTGGAGTTCAGACGTGTGCTCTTCCGATCT
Reverse index 9 adapter primer	CAAGCAGAAGACGGCATACGAGATCTGATCGTGACTGGAGTTCAGACGTGTGCTCTTCCGATCT
Reverse index 10 adapter primer	CAAGCAGAAGACGGCATACGAGATAAGCTAGTGACTGGAGTTCAGACGTGTGCTCTTCCGATCT

Sample index barcode index are shown in red.

Additional References

[74] R.E. Board, V.S. Williams, L. Knight, J. Shaw, A. Greystoke, M. Ranson, C. Dive, F.H. Blackhall, A. Hughes, Isolation and Extraction of Circulating Tumor DNA from Patients with Small Cell Lung Cancer, Ann. N. Y. Acad. Sci. 1137 (2008) 98–107. doi:10.1196/annals.1448.020.
